# Use of a b-Value of 3000 s/mm^2^ to Differentiate Benign from Malignant Hepatic Masses in Liver Diffusion-Weighted Imaging Using a 3T MR Scanner with a Peak Gradient of 100 mT/m

**DOI:** 10.14789/ejmj.JMJ25-0005-OA

**Published:** 2025-07-31

**Authors:** KATSUHIRO SANO, KEITA FUKUSHIMA, HARUHIKO MACHIDA, TOSHIYA KARIYASU, HIROSHI KUSAHARA, SANAE TAKAHASHI, AKIHITO NAKANISHI, KENICHI YOKOYAMA

**Affiliations:** 1 Department of Radiology, Juntendo University Graduate School of Medicine, Tokyo, Japan; 1 Department of Radiology, Juntendo University Graduate School of Medicine, Tokyo, Japan; 2 Department of Radiology, Faculty of Medicine, Kyorin University, Tokyo, Japan; 2 Department of Radiology, Faculty of Medicine, Kyorin University, Tokyo, Japan; 3 Department of Radiology, Kyorin University Hospital, Tokyo, Japan; 3 Department of Radiology, Kyorin University Hospital, Tokyo, Japan; 4 Department of Radiology, Tokyo Women’s Medical University, Adachi Medical Center, Tokyo, Japan; 4 Department of Radiology, Tokyo Women’s Medical University, Adachi Medical Center, Tokyo, Japan; 5 MRI Systems Division, Canon Medical Systems Corporation, Tochigi, Japan; 5 MRI Systems Division, Canon Medical Systems Corporation, Tochigi, Japan

**Keywords:** magnetic resonance imaging, diffusion-weighted imaging, liver tumor, peak gradient of 100 mT/m, a b-value of 3000 s/mm^2^

## Abstract

**Objectives:**

This study aimed to evaluate the clinical utility of a b-value of 3000 s/mm^2^ in liver diffusion-weighted imaging (DWI) for differentiating between benign and malignant hepatic masses using a 3T MR scanner with a peak gradient of 100 mT/m.

**Materials:**

Fifty-four patients (33 men, 21 women; mean age: 65 years) with hepatic masses, including 13 hepatocellular carcinomas (HCCs), 18 hepatic metastases, 12 hepatic hemangiomas, and 11 hepatic cysts, were prospectively enrolled.

**Methods:**

Liver DWI was performed at b-values of 1000 and 3000 s/mm^2^. Quantitative analyses included signal-to-noise ratio (SNR), contrast ratio (CR), and apparent diffusion coefficient (ADC). Two independent readers assessed qualitative signal intensity (SI) scores of hepatic masses. The diagnostic performance for differentiating between benign and malignant hepatic masses was evaluated using receiver operating characteristic (ROC) analysis and compared between the two b-values.

**Results:**

A b-value of 3000 s/mm^2^ provided significantly higher AUCs for SNR, CR, and SI scores than 1000 s/mm^2^ (P < 0.05). The SI score at 3000 s/mm^2^ achieved an AUC of 1.00, with 100% sensitivity and specificity. While malignant masses maintained high SI across both b-values, benign masses showed significantly lower SI at 3000 s/mm^2^ (P < 0.001). ADC values were significantly lower at 3000 s/mm^2^.

**Conclusions:**

Liver DWI at a b-value of 3000 s/mm^2^ enhances diagnostic accuracy in differentiating hepatic masses. The use of this higher b-value preserves the high SI of malignancies while effectively reducing false positives from the T2 shine-through effect, making it a valuable imaging approach for clinical applications.

## Introduction

Visual evaluation of diffusion-weighted imaging (DWI) with apparent diffusion coefficient (ADC) measurement is widely used in brain magnetic resonance (MR) examinations to assess conditions such as acute cerebral ischemic stroke, intracranial tumors, and demyelinating diseases. In liver DWI, artifacts caused by respiratory and cardiac motion have been reduced with the advent of echo-planar and parallel imaging sequences. Since then, DWI has been commonly employed to distinguish between benign and malignant hepatic masses at b-values of 800-1000 s/mm^2^, using both 1.5-Tesla (1.5T) and 3.0-Tesla (3T) MR scanners^1-3)^. However, the utility of visual evaluation for liver DWI remains limited, even at higher b-values, as some benign hepatic masses, such as hepatic hemangiomas, can exhibit high signal intensity (SI) similar to malignant hepatic masses due to the T2 shine-through effect^1, 2, 4, 5)^. A b-value of ≥ 2000 s/mm^2^ has been shown to improve sensitivity and specificity in detecting malignant masses in MR examinations of the brain, prostate, and other regions by reducing the T2 shine-through effect^3-9)^. However, no previous studies have demonstrated the usefulness of DWI with b-values above 1500 s/mm^2^ in the liver, including computed DWI (cDWI), as its diagnostic performance tends to be compromised by image quality degradation^10-12)^.

A state-of-the-art 3T MR scanner with a peak gradient of 100 mT/m has recently been introduced in clinical practice. This scanner is designed to shorten the echo time (TE) and achieve a sufficiently high signal-to-noise ratio (SNR) in liver DWI, even at a b-value of 3000 s/mm^2^, by increasing the amplitude of motion-probing gradient pulses and reducing the application time of these pulses. Fukushima et al.^5)^ used this scanner with a minimal TE of 53 ms and a b-value of 3000 s/mm^2^ to obtain diagnostic image quality and mitigate the T2 shine-through effect, thereby reducing false-positive detection of benign hepatic masses, such as hepatic hemangiomas, in liver DWI. Diagnosing typical hemangiomas using abdominal ultrasound, multiphase contrast-enhanced computed tomography (CT), and dynamic contrast-enhanced MR with conventional extracellular gadolinium-based contrast agents is generally straightforward, except in the case of certain atypical hemangiomas^13, 14)^. However, differentiating between benign hepatic masses, including hemangiomas, and malignant masses remains a critical challenge in daily liver MR examinations, even with the increased use of hepatocyte-specific contrast agents^15, 16)^. Therefore, this prospective clinical pilot study aimed to assess the clinical utility of a b-value of 3000 s/mm^2^ in liver DWI using a 3T MR scanner to differentiate between benign and malignant hepatic masses.

## Materials and Methods

### Study approval and consent

This prospective, single-institution study was approved by the institutional review board (Approval Number: H27-005-09, March 3, 2020) and conducted in accordance with the principles of the Declaration of Helsinki. Written informed consent was obtained from all patients before participation.

### Patient selection

The inclusion criteria for this study were as follows: (1) adult patients with hepatic masses detected via ultrasound and/or CT; (2) patients who underwent liver MR for differential diagnosis, including gadoxetic acid-enhanced MR and DWI performed at b-values of 1000 and 3000 s/mm^2^ with a minimal TE from October 2018 to April 2019; and (3) patients with hepatic masses measuring ≥ the maximum diameter of 5 mm.

**Figure 1:**
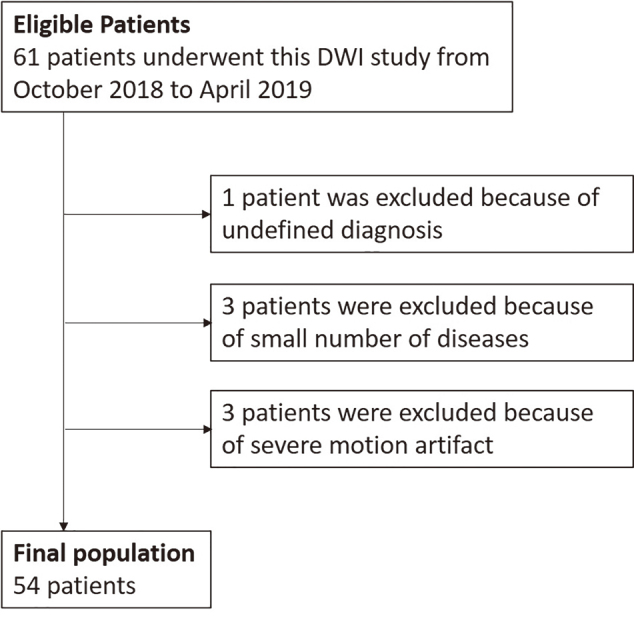
Flowchart of study population

Exclusion criteria included (1) a small number of rare hepatic disease cases (e.g., hepatic abscess, complicated cyst, or focal nodular hyperplasia) and (2) severe motion artifacts on DWI. Of the 61 patients who underwent liver DWI at b-values of 1000 and 3000 s/mm^2^, seven patients were excluded for the following reasons: the absence of a definitive diagnosis (n = 1), the presence of rare conditions (hepatic abscess, complicated cyst, or focal nodular hyperplasia, n = 1 each), and severe motion artifacts (n = 3). Thus, 54 patients (33 men and 21 women; mean age: 65 ± 14 years; range: 38-85 years; mean body weight: 61.1 ± 12.6 kg; range: 44-96 kg) were included in the study (Figure 1). Only the largest hepatic mass was included for each patient. In cases where both benign and malignant hepatic masses were present, only the largest malignant mass was evaluated. A total of 54 hepatic masses were classified as follows: 13 cases (24%) of hepatocellular carcinomas (HCCs), 18 cases (33%) of hepatic metastasis, 12 cases (22%) of hepatic hemangioma, and 11 cases (21%) of hepatic cyst. The size distribution of the hepatic masses was as follows: less than 10 mm in 1 case (2%), 10-20 mm in 24 cases (44%), and greater than 20 mm in 29 cases (54%). The overall average size was 29.0 ± 20.5 mm, with a range of 5-105 mm. The masses were distributed among the liver segments as follows: segment 1 (n = 2), segment 2 (n = 4), segment 3 (n = 4), segment 4 (n = 7), segment 5 (n = 3), segment 6 (n = 14), segment 7 (n = 9), and segment 8 (n = 11).

### Definitive diagnosis of hepatic masses

Nine HCCs were diagnosed pathologically after liver resection surgery, while one was confirmed through liver biopsy. Among the HCCs, one was well-differentiated, five were moderately differentiated, and four were poorly differentiated. A board- certified abdominal radiologist with 18 years of experience (KS) diagnosed the remaining three HCCs as category LR-5, according to the 2018 Liver Imaging Reporting and Data System (LI- RADS) criteria^17)^. Of the 18 hepatic metastases, primary malignancies were colorectal carcinoma (n = 11), pancreatic carcinoma (n = 3), gastric carcinoma (n = 2), and ovarian carcinoma (n = 2). Twelve hepatic metastases were diagnosed pathologically following liver resection, and three were confirmed through liver biopsy. For the remaining three cases, diagnosis was based on contrast-enhanced CT, which revealed peripheral rim enhancement with an increase in diameter of at least 20% during serial imaging follow-up in patients with known primary malignancies^18)^. Hepatic hemangiomas were diagnosed based on the following imaging characteristics: well-demarcated low SI on T1-weighted imaging (T1WI), high SI on T2-weighted imaging (T2WI), and peripheral globular discontinuous enhancement ("bright-dot enhancement") with progressive centripetal enhancement or "delayed fill-in" on dynamic contrast-enhanced T1WI or multiphasic contrast-enhanced CT. Hepatic cysts were characterized by well-demarcated homogeneous low SI on T1WI, high SI on T2WI, and the absence of post-contrast enhancement on T1WI or CT.

### DWI acquisition

All patients underwent liver DWI using a 3T MR scanner (Vantage Galan 3T/ZGO; Canon Medical Systems, Tochigi, Japan) equipped with a phased-array coil. The scans were performed using a spin-echo echo-planar imaging sequence with real-time respiratory motion correction (Atlas SPEEDER Body & Spine Coil, Canon Medical Systems). DWI was acquired at b-values of 0, 1000, and 3000 s/mm^2^, with minimal TEs of 44 ms for the b-value of 1000 s/mm^2^ and 53 ms for the b-value of 3000 s/mm^2^. Other acquisition parameters were: repetition time (TR) = 3500 ms, flip angle = 90°/180°, matrix size = 160 × 160, field of view (FOV) = 33 × 36 cm, slice thickness/gap = 6/0 mm, and parallel imaging factor (SPEEDER, Canon Medical Systems) = 2. Fat saturation was achieved using the spectral attenuated inversion recovery technique. The average acquisition times for DWI at b-values of 1000 and 3000 s/mm^2^ were 176 ± 35 s and 180 ± 37 s, respectively. DWI at both b-values was acquired during the waiting time before the hepatobiliary phase of gadoxetic acid-enhanced MR, following the administration of gadoxetic acid (gadolinium- ethoxybenzyl-diethylenetriamine; Primovist, Bayer Pharma Japan, Osaka, Japan).

### Quantitative image evaluation

A board-certified abdominal radiologist (KS) and a radiological technologist (KF) with 13 years of MR experience conducted quantitative measurements for each patient. Referring to the DWI at a b-value of 0 s/mm^2^, they placed a circular region of interest (ROI) of maximum size within the hepatic mass and a 2-cm^2^ ROI within the liver parenchyma near the hepatic mass, avoiding the intrahepatic bile ducts and vessels, in both DWI sets acquired at b-values of 1000 and 3000 s/mm^2^. The position of the ROI was verified by another board-certified radiologist (TK) with similar expertise. The SI of the hepatic mass (SI_M_) and the liver parenchyma (SI_P_) was recorded for both b-values. A circular ROI at 2 cm^2^ was placed in the air near the body surface to measure its standard deviation (SD) as the background noise. Images of DWI at b-value of 0 s/mm^2^ were used for this ROI placement as the reference when the hepatic masses were not visualized on DWI at b-values of 1000 and 3000 s/mm^2^.

The SNR was calculated as SI_M_ divided by the SD of the background in DWI at both b-values. Additionally, the contrast ratio (CR) was calculated as SI_M_/SI_P_ at both b-values. Additionally, the ADC of the mass was calculated using the following equation: ADC = −ln [(SI (*b*1)/SI (*b*0)]/(*b*1 − *b*0), where b0 = 0 and b1 = 1000 or 3000 s/mm^2^^5)^. This equation provides the ADC value for each patient based on the DWI acquired at both b-values. The mean values for SNR, CR, and ADC were computed at both b-values for each hepatic mass.

### Qualitative image evaluation

**Figure 2:**
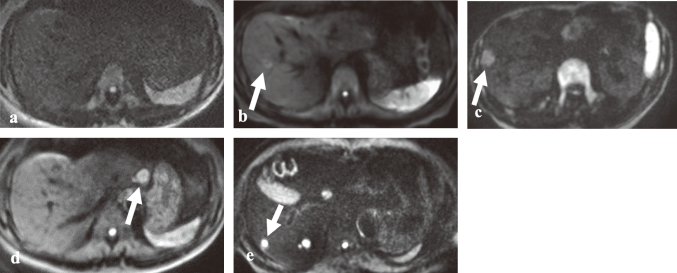
Five-point scale for qualitative image evaluation Representative images of hepatic masses in liver diffusion-weighted imaging (DWI) illustrate the five-point scale: Point 1 (a): Not visible; Point 2 (b): Slightly visible; Point 3 (c): Visible, but with lower signal intensity (SI) compared to the spleen; Point 4 (d): Clearly visible, with SI similar to the spleen; Point 5 (e): Very clearly visible, with high SI similar to the spinal cord.

Two independent readers, consisting of a board- certified abdominal radiologist with 15 years of experience (TK) and a radiological technologist (KF), evaluated the SI of hepatic masses on liver DWI at b-values of 1000 and 3000 s/mm^2^. The evaluation was conducted using a five-point scale, defined as follows: 1: Not visible; 2: Slightly visible; 3: Visible, but with lower SI compared to the spleen; 4: Clearly visible, with SI similar to the spleen; 5: Very clearly visible, with high SI similar to the spinal cord. Figure 2 provides representative images corresponding to each of these points on the scale. The readers, blinded to the imaging parameters, scored the visibility of the hepatic masses at both b-values based on this five-point system.

Additionally, the readers qualitatively assessed the signal and noise of the liver parenchyma in both lobes on DWI at b-values of 1000 and 3000 s/mm^2^, using a three-point scale: 1 (Poor): Nondiagnostic due to either no apparent signal or severe noise; 2 (Moderate): Diagnostic, but with insufficient signal and indistinct contours or moderate noise; 3 (Excellent): Sufficient signal, distinct contours, and no significant noise.

### Statistical analysis

All continuous variables were expressed as mean ± SD, while categorical variables were expressed as median and interquartile range (IQR). The comparison of SNR, CR, ADC, and qualitative SI scores of the hepatic masses, as well as the qualitative SI scores of the hepatic parenchyma, between the two b-values (1000 and 3000 s/mm^2^) was performed using the Wilcoxon signed-rank test.

Inter-reader agreement for the qualitative SI scores of hepatic masses and hepatic parenchyma was evaluated using weighted kappa statistics. Kappa values were interpreted as follows: 0.81-1.00, Excellent agreement; 0.61-0.80, Substantial agreement; 0.41-0.60, Moderate agreement; 0.21-0.40, Fair agreement; and 0.00-0.20, Poor agreement.

The diagnostic accuracy of both readers in distinguishing between malignant and benign hepatic masses was assessed using receiver operating characteristic (ROC) analysis. For this analysis, SI scores of 4 and 5 were considered positive (indicating malignancy), while SI scores of 1 to 3 were considered negative (indicating benignity). The area under the curve (AUC) was calculated to quantify the diagnostic performance. The AUCs were compared between the two b-values (1000 and 3000 s/mm^2^) using the Wilcoxon signed-rank test^19, 20)^. Sensitivity, specificity, and cutoff values were also calculated based on the ROC analysis for SNR, CR, ADC, and qualitative SI scores.

All statistical analyses were performed using commercially available software: JMP version 14.2 (SAS Institute, Japan) and SPSS version 29.0.0.0 (IBM Japan, Tokyo, Japan). A P-value of less than 0.05 was considered statistically significant.

## Results

### Comparison between the different b-values

The SNR for all types of hepatic masses at a b- value of 1000 s/mm^2^ was significantly higher than at 3000 s/mm^2^ (P ≤ 0.001), as shown in Table 1. The CR values for HCCs, hepatic metastases, and hepatic hemangiomas were comparable between the two b-values (P = 0.718, 0.086, and 0.053, respectively). However, the CR for hepatic cysts was significantly lower at a b-value of 3000 s/mm^2^ than at 1000 s/mm^2^ (P = 0.002). The ADC values for all mass types at 3000 s/mm^2^ were significantly lower compared to 1000 s/mm^2^ (P < 0.001 for HCCs, metastases, and hemangiomas; P = 0.003 for cysts).

The qualitative SI score for malignant hepatic masses (HCCs and hepatic metastases) was comparable between both b-values for both readers (P- values for Reader 1: 1.000 and 1.000, respectively; Reader 2: 1.000 and 0.625, respectively), as shown in Table 2. In contrast, the SI score for benign hepatic masses (hemangiomas and cysts) was significantly lower at a b-value of 3000 s/mm^2^ compared to 1000 s/mm^2^ for both readers (P < 0.001 and 0.002, respectively). All malignant hepatic masses had SI scores of 4 or 5 at a b-value of 3000 s/mm^2^, while benign hepatic masses had scores ≤ 3.

**Figure 3:**
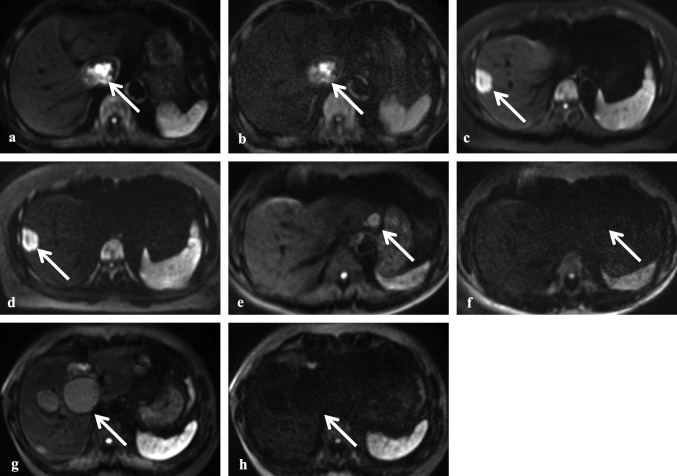
Representative diffusion-weighted imaging (DWI) appearance of four different hepatic mass types at b-values of 1000 and 3000 s/mm^2^ Representative liver DWI images of four different hepatic mass types at b-values of 1000 and 3000 s/mm^2^: a, b: A 78-year-old man with hepatocellular carcinoma (HCC); c, d: A 47-year-old woman with hepatic metastasis from rectal carcinoma; e, f: A 57-year-old man with hepatic hemangioma; g, h: A 66-year-old woman with a hepatic cyst. a, c, e, g show liver DWI at a b-value of 1000 s/mm^2^, while b, d, f, h show DWI at a b-value of 3000 s/mm^2^. The HCC (a, b) and hepatic metastasis (c, d) exhibit high signal intensity (SI) similar to or greater than that of the spleen at both b-values. In contrast, the hepatic hemangioma (e, f) and cyst (g, h) are visible with SI similar to or less than that of the spleen at 1000 s/mm^2^ (e, g) but are not visible at 3000 s/mm^2^ (f, h).

Figure 3 provides representative DWI images of the different mass types at b-values of 1000 and 3000 s/mm^2^. HCC and hepatic metastasis showed SI similar to or higher than the spleen at both b-values, with high SI being better preserved at 3000 s/mm^2^ compared to 1000 s/mm^2^. In contrast, hepatic hemangiomas and cysts were visible with SI similar to or lower than the spleen at a b-value of 1000 s/mm^2^ but were not visible at 3000 s/mm^2^. The SI reduction was more pronounced at 3000 s/mm^2^ compared to 1000 s/mm^2^. Inter-reader agreement for the qualitative SI scores was excellent at both b-values (κ = 0.97 for 1000 s/mm^2^ and κ = 0.99 for 3000 s/mm^2^), as shown in Table 2.

The qualitative SI scores of hepatic parenchyma were significantly higher at 1000 s/mm^2^ than at 3000 s/mm^2^ for both readers (P < 0.001), as shown in Table 3. Inter-reader agreement was excellent at 1000 s/mm^2^ (κ = 0.90) and substantial at 3000 s/mm^2^ (κ = 0.80) (Table 3).

**Table 1:** SNR, CR, and ADC of hepatic masses

	b = 1000 s/mm^2^	b = 3000 s/mm^2^	P value
SNR			
HCC	244.80 ± 187.10	65.46 ± 22.20	< 0.001
Metastasis	271.93 ± 126.16	87.85 ± 44.95	< 0.001
Hemangioma	204.94 ± 108.60	42.09 ± 25.62	< 0.001
Cyst	112.84 ± 57.02	20.17 ± 13.92	0.001
CR			
HCC	2.84 ± 1.28	3.00 ± 1.60	0.718
Metastasis	3.16 ± 1.29	3.77 ± 1.74	0.086
Hemangioma	1.84 ± 0.60	1.57 ± 0.56	0.053
Cyst	2.29 ± 1.35	1.04 ± 0.35	0.002
ADC (× 10^−3^ mm^2^/s)			
HCC	1.06 ± 0.31	0.73 ± 0.15	< 0.001
Metastasis	1.04 ± 0.20	0.72 ± 0.15	< 0.001
Hemangioma	1.75 ± 0.45	1.04 ± 0.21	< 0.001
Cyst	2.32 ± 0.68	1.42 ± 0.24	0.003

ADC: apparent diffusion coefficient; CR: contrast ratio; HCC: hepatocellular carcinoma; SNR: signal to noise ratio. Data are expressed as mean ± standard deviation.

**Table 2:** Qualitative SI score of hepatic masses

	b = 1000 s/mm^2^	b = 3000 s/mm^2^	P value
Reader 1			
HCC	5 (4.5-5)	5 (4.5-5)	1.000
Metastasis	5 (5-5)	5 (5-5)	1.000
Hemangioma	4.5 (4-5)	2 (1-3)	< 0.001
Cyst	3 (2-3)	1 (1-1)	0.002
Reader 2			
HCC	5 (4.5-5)	5 (4.5-5)	1.000
Metastasis	5 (5-5)	5 (5-5)	0.625
Hemangioma	4.5 (4-5)	2 (1-3)	< 0.001
Cyst	3 (3-3)	1 (1-1)	0.002
κ value	0.97*	0.99*	

HCC: hepatocellular carcinoma; SI: signal intensity. Data are expressed as median (interquartile range). Asterisks represent an excellent inter-reader agreement.

**Table 3:** Qualitative SI score of hepatic parenchyma

	b = 1000 s/mm^2^	b = 3000 s/mm^2^	P value
Reader 1			
Right lobe	3 (3-3)	2 (2-2)	< 0.001
Left lobe	2 (2-3)	2 (2-2)	< 0.001
Reader 2			
Right lobe	3 (3-3)	2 (2-2)	< 0.001
Left lobe	2 (2-3)	2 (2-2)	< 0.001
κ value	0.90*	0.80*	

SI: signal intensity. Data are expressed as median (interquartile range). Asterisks represent an excellent and substantial inter-reader agreement.

### Differentiation between benign and malignant hepatic masses

The SNR (P = 0.002 for b = 1000 s/mm^2^ and P < 0.001 for b = 3000 s/mm^2^), CR (P < 0.001 for both), and qualitative SI score (P < 0.001 for all) of malignant hepatic masses were significantly higher than those of benign masses at both b-values of 1000 and 3000 s/mm^2^ for both readers, as shown in Table 4. The ADC of malignant hepatic masses was significantly lower than that of benign masses at both b-values (P < 0.001). ROC curve analysis revealed that the AUC, sensitivity, and specificity of ADC for differentiating between malignant and benign hepatic masses were comparable between the two b-values (P = 0.517). In contrast, the AUC, sensitivities, and specificities of SNR, CR, and qualitative SI score for differentiating between malignant and benign hepatic masses were significantly higher at b = 3000 s/mm^2^ than at b = 1000 s/mm^2^ (P = 0.002, 0.007, 0.002, respectively).

Notably, perfect differentiation (AUC: 1.00, sensitivity: 100%, specificity: 100%) was achieved by both readers using the qualitative SI score with a cutoff value of 4 at b = 3000 s/mm^2^. The high signal intensity of malignant masses was maintained at b = 3000 s/mm^2^, allowing for accurate differentiation from benign masses, as demonstrated in Figure 3.

**Table 4:** Differentiation between benign and malignant hepatic masses at b-values of 1000 and 3000 s/mm^2^

	Benign	Malignant	P value	AUC	Cutoff value	Sensitivity	Specificity
SNR							
b = 1000 s/mm^2^	160.89 ± 97.92	260.55 ± 152.34	0.002	0.75	212.50	21/31 (68%)	18/23 (79%)
b = 3000 s/mm^2^	31.61 ± 23.28	78.46 ± 33.32	< 0.001	0.89	35.60	31/31 (100%)	17/23 (74%)
P value	< 0.001	< 0.001		0.002			
CR							
b = 1000 s/mm^2^	2.06 ± 1.03	3.03 ± 1.27	< 0.001	0.79	2.10	24/31 (77%)	16/23 (70%)
b = 3000 s/mm^2^	1.31 ± 0.53	3.45 ± 1.70	< 0.001	0.95	1.60	31/31 (100%)	18/23 (78%)
P value	< 0.001	0.136		0.007			
ADC (× 10^-^^3^)							
b = 1000 s/mm^2^	2.02 ± 0.63	1.05 ± 0.25	< 0.001	0.93	1.37	28/31 (90%)	20/23 (87%)
b = 3000 s/mm^2^	1.52 ± 0.79	0.72 ± 0.15	< 0.001	0.95	0.92	29/31 (94%)	20/23 (87%)
P value	< 0.001	< 0.001		0.517			
Qualitative SI score (Reader 1)							
b = 1000 s/mm^2^	4 (3-5)	5 (5-5)	< 0.001	0.84	5.0	27/31 (87%)	17/23 (74%)
b = 3000 s/mm^2^	1 (1-2)	5 (5-5)	< 0.001	1.00	4.0	31/31 (100%)	23/23 (100%)
P value	< 0.001	1.000		0.002			
Qualitative SI score (Reader 2)							
b = 1000 s/mm^2^	4 (3-5)	5 (5-5)	< 0.001	0.84	5.0	27/31 (87%)	17/23 (74%)
b = 3000 s/mm^2^	1 (1-2)	5 (5-5)	< 0.001	1.00	4.0	31/31 (100%)	23/23 (100%)
P value	< 0.001	0.423		0.002			

ADC: apparent diffusion coefficient; AUC: area under curve; CR: contrast ratio; SI: signal intensity; SNR: signal to noise ratio. Data are expressed as mean ± standard deviation or median (interquartile range).

## Discussion

This study evaluated the clinical utility of a b- value of 3000 s/mm^2^ in liver DWI for accurately differentiating between benign and malignant hepatic masses using a 3T MR scanner with a peak gradient of 100 mT/m. Notably, the qualitative SI score for benign hepatic masses was significantly lower at 3000 s/mm^2^ compared to 1000 s/mm^2^ for both readers, while the qualitative SI score for malignant hepatic masses remained comparable across both b-values. Inter-reader agreement ranged from substantial to excellent. The diagnostic accuracy of the SI score was significantly higher at a b-value of 3000 s/mm^2^ than at 1000 s/mm^2^. The SI score outperformed both the CR and ADC, and achieved perfect results (AUC: 1.00; sensitivity: 100%; specificity: 100%) with a cutoff value of 4 at a b-value of 3000 s/mm^2^.

DWI using 1.5T and 3T MR scanners has commonly been applied to differentiate between benign and malignant hepatic masses at b-values of 800-1000 s/mm^2^^1-3, 5)^. However, the utility of visual assessment in liver DWI remains limited for this purpose, even at these b-values, as some benign hepatic masses may exhibit high SI due to the T2 shine-through effect^9, 14)^. Although ADC measurements have improved differentiation, they remain susceptible to factors such as image noise^18-21)^. Furthermore, significant overlap in ADC values between benign and malignant masses has been reported, which limits consistent differentiation^3, 21-26)^. The use of a minimal TE of 53 ms and a b-value of 3000 s/mm^2^ with our scanner has been shown to reduce the T2 shine-through effect while preserving diagnostic image quality in liver DWI^5)^. ADC provides information about both diffusion and perfusion. This study revealed that ADC values were significantly lower for all mass types at a b-value of 3000 s/mm^2^ compared to 1000 s/mm^2^, suggesting that ADC at 3000 s/mm^2^ may better represent the true diffusion coefficient with fewer perfusion effects. As a result, the CR of hepatic cysts was significantly lower at a b-value of 3000 s/mm^2^ than at 1000 s/mm^2^, while the CR for hepatic hemangiomas remained comparable. The SI score of benign hepatic masses was significantly lower at a b-value of 3000 s/mm^2^, which may improve specificity in detecting malignant hepatic masses with high cellularity. Consequently, visual SI comparable to or greater than that of the spleen allowed for the perfect distinction of malignant hepatic masses from benign ones, particularly distinguishing hepatic metastases from hepatic hemangiomas. This diagnostic approach can improve the accuracy of liver DWI compared to previous meta-analyses, which reported AUC values of 0.95-0.96 in differentiating between benign and malignant hepatic masses^27-29)^. In clinical practice, distinguishing between hepatic metastases and hemangiomas with poor contrast enhancement can be challenging, even with contrast-enhanced dynamic imaging. Therefore, this diagnostic approach may assist in their differentiation.

cDWI presents a viable alternative to our method. The TE used for cDWI is identical to the minimal TE required to acquire DWI at a b-value of 1000 s/mm^2^, which is shorter than the minimal TE for acquiring DWI at a b-value of 3000 s/mm^2^, as cDWI at 3000 s/mm^2^ is calculated using DWI acquired at 1000 s/mm^2^^4, 5)^. In theory, malignant hepatic masses may be more easily visualized using cDWI at a b-value of 3000 s/mm^2^ compared to actual DWI at the same b-value, due to their higher SI. Conversely, benign hepatic masses, such as hepatic hemangiomas, may become more visible than desired, owing to an increased T2 shine-through effect.

This study has certain limitations. First, the minimal TE varied between b-values of 1000 and 3000 s/mm^2^ using our MR scanner. However, using the minimal TE maximized the SNR of the hepatic parenchyma, improved image quality, and reduced DWI acquisition time at each b-value^5)^. Second, HCCs and hepatic metastases are the most common hepatic masses; therefore, we only included these as malignant masses, while hepatic hemangiomas and cysts were included as benign masses. Other hepatic masses, such as intrahepatic cholangiocarcinoma, focal nodular hyperplasia, and hepatic abscess, were excluded due to the small number of patients in this study. Notably, the inclusion of patients with hepatic abscesses might significantly alter the results, as abscesses can show restricted diffusion, similar to malignant masses, due to their high viscosity. Furthermore, if abscesses or other lesions with restricted diffusion are included in future studies, the diagnostic performance of DWI at a b-value of 3000 s/mm^2^ could be decrease. Third, we included various secondary malignancies, such as ovarian carcinomas may behave differently from colorectal, pancreatic, and gastric carcinomas. Fourth, the body weight of our Japanese patients was lower than that of average-sized patients in Western countries, where the SNR of the hepatic parenchyma in liver DWI tends to decrease. Fifth, this study included only a small number of hepatic masses in the left lobe. The incidence of non-diagnostic image quality may increase if more masses from the left lobe are included, as the SNR of this lobe in liver DWI is susceptible to cardiac motion, particularly at a b-value of 3000 s/mm^2^. However, Fukushima et al. used the same MR scanner and showed that overall image quality in liver DWI was comparable between the b-value of 1000 s/mm^2^ with routine TE and the b-value of 3000 s/mm^2^ with minimal TE^5)^. The overall image quality was diagnostic at both b-values. Sixth, while the image quality of DWI at 3000 s/mm^2^ was not better than at 1000 s/mm^2^ due to lower SNR, our results indicate that DWI at 3000 s/mm^2^ still provided diagnostic image quality. We could not assess whether smaller-sized nodules might be missed with DWI at 3000 s/mm^2^, as there were few masses < 1 cm in our study. If the number of nodules smaller than 1 cm increases, the lesion detection sensitivity of DWI at a b-value of 3000 s/mm^2^ could decrease. Seventh, the optimal high b-value for liver imaging has not yet been established, and it remains unclear whether 3000 s/mm^2^ is the ideal choice. If similar results can be obtained using b-values in the range of 1500-2500 s/mm^2^, it may suggest that a b-value with better SNR exists. Thus, further studies are needed to determine the optimal b-value for liver DWI that best balances diagnostic accuracy and image quality. Eighth, in some cases, a pathological diagnosis was not obtained. Ninth, this study did not employ noise reduction techniques, such as deep learning reconstruction, which might have improved image quality. This is a subject for future investigation. Tenth, this study did not consider non-Gaussian diffusion. Therefore, future studies should explore approaches such as diffusion kurtosis imaging. Finally, this prospective study included a small patient population from a single institution. Additional research involving a larger cohort from multiple institutions is necessary to validate our findings.

In conclusion, a b-value of 3000 s/mm^2^ in liver DWI using a state-of-the-art 3T MR scanner with a peak gradient of 100 mT/m is clinically more effective in differentiating between benign and malignant hepatic masses compared to a b-value of 1000 s/mm^2^. The results of this study demonstrated that with DWI at a b-value of 3000 s/mm^2^, the high signal of malignancy was preserved, while the signal from the T2 shine-through effect in hemangiomas was reduced. These findings suggest that the optimal b-value for liver DWI on a 3T MR scanner with a peak gradient of 100 mT/m is higher than the conventional 800-1000 s/mm^2^.

## Funding

The authors received no financial support for the research.

## Author contributions

KS, KF, HM, and TK jointly supervised the study, conceived the research, collected data, performed data analysis, and primarily contributed to manuscript writing. HK contributed to establishing the MRI acquisition method. ST contributed to data collection. All authors read and approved the final manuscript.

## Conflicts of interest statement

Katsuhiro Sano, Keita Fukushima, Haruhiko Machida, Toshiya Kariyasu, Sanae Takahashi, Akihito Nakanishi, and Kenichi Yokoyama have no conflicts of interest. Hiroshi Kusahara is employed by Canon Medical Systems Corporation.
